# Adolescents’ Empowerment for Mental Health Literacy in School: A Pilot Study on ProLiSMental Psychoeducational Intervention

**DOI:** 10.3390/ijerph18158022

**Published:** 2021-07-29

**Authors:** Tânia Morgado, Luís Loureiro, Maria Antónia Rebelo Botelho, Maria Isabel Marques, José Ramón Martínez-Riera, Pedro Melo

**Affiliations:** 1 Centro Hospitalar e Universitário de Coimbra—Hospital Pediátrico, Av. Afonso Romão, 3000-062 Coimbra, Portugal; 2 Health Sciences Research Unit: Nursing, Escola Superior de Enfermagem de Coimbra, Av. Bissaya Barreto, 3004-011 Coimbra, Portugal; luisloureiro@esenfc.pt (L.L.); imarques@esenfc.pt (M.I.M.); 3 NursID: Innovation & Development in Nursing, Center for Health Technology and Services Research, 4200-450 Porto, Portugal; 4 Escola Superior de Enfermagem de Coimbra, Av. Bissaya Barreto, 3004-011 Coimbra, Portugal; 5 Escola Superior de Enfermagem de Lisboa, Av. Dom João II, Lote 4.69.01, 1990-096 Lisboa, Portugal; rbotelho@esel.pt; 6 Departamento Enfermeria Comunitaria, Medicina Preventiva y Salud Publica e Historia de la Ciencia, Universidad de Alicante, E-03080 Alicante, Spain; jr.martinez@ua.es; 7 Centre for Interdisiplinary Research in Health, Universidade Católica Portuguesa, 4169-005 Porto, Portugal; pmelo@porto.ucp.pt

**Keywords:** adolescent, health literacy, health education, mental health, anxiety, mental health nursing, school nursing, pilot study

## Abstract

Adolescence is a critical life phase for mental health and anxiety an emerging challenge for adolescents. Psychoeducational interventions to promote mental health literacy (MHL) on anxiety in adolescents are needed. This study aimed to test the primary outcome of a future full-scale trial: improvement of adolescents’ anxiety MHL components on recognition, prevention strategies, and self-help strategies. A sample of 38 adolescents, 24 (63.2%) females and 14 (36.8%) males, with an average age of 14.50 years (SD = 0.89) participated in this study. Each class was allocated to the intervention group (IG, *n* = 21) or the waiting list control group (WLCG, *n* = 17) with single-blinded randomization. MHL was assessed using the QuALiSMental. The ProLiSMental psychoeducational intervention consists of four or eight weekly sessions of 90 or 45 min for adolescents, using different active pedagogical methods and techniques. There also are initial and final sessions with adolescents, legal guardians, and teachers. There was a significant improvement with a small to relatively strong effect size in many dimensions of anxiety MHL components. This study suggests the progression to the full-scale trial and values the important role of mental health and psychiatric nurses in the adolescents’ empowerment for MHL in schools.

## 1. Introduction

The COVID 19 pandemic had implications for many dimensions of population health, including the mental health of people throughout the life cycle. In adolescents, several articles report the effects of the pandemic on their mental health [1,2,3,4,5,6], with approximately 38% of children and adolescents experiencing anxiety in the studies found in the systematic review carried out by Octavius et al. (2020) [5]. Some qualitative [7] and quantitative studies carried out in Portugal also report this reality [8,9]. Orgilés et al. (2021) [9] refer that the prevalence of anxiety and depressive symptoms was higher than usual and in the Portuguese sample, a prevalence of 26.5% was found, slightly higher than the prevalence (11.5%) informed by Gaspar de Matos, Barrett, Dadds, and Shortt (2003) [10].

The “WHO Special Initiative for Mental Health (2019–2023): Universal Health Coverage for Mental Health” intends to empower communities and individuals to attain the highest standard of health, which can only be achieved when their mental health and well-being are ensured [11]. Interventions to promote adolescents’ mental health and mental health literacy (MHL) on anxiety and to prevent pathological anxiety are necessary.

In the same way, health literacy (HL) is a population strategy for health promotion and public health [12,13], MHL is a significant strategy for mental health promotion, empowering the community to take action for better mental health [14,15] and promoting community mental health. The MHL concept was defined in the late 1990s as the “knowledge and beliefs about mental disorders which aid their recognition, management, and prevention” [16]. In the 2000s, components of the concept of literacy were defined. These components consist of: (a) knowledge of how to prevent mental disorders; (b) recognition of when a disorder is developing; (c) knowledge of help-seeking options and treatments available; (d) knowledge of effective self-help strategies for milder problems; (e) first aid skills to support others who are developing a mental disorder or are in a mental health crisis [14]. More recently, the concept has focused more on “understanding how to obtain and maintain positive mental health” [17]. Bjørnsen, Eilertsen, Ringdal, Espnes, and Moksnes (2017) referred that this conceptualization advances previous perceptions of MHL as merely knowledge of mental disorders and is in line with the WHO’s definition of mental health (Organization, 2013) and proposed the term “positive mental health literacy” [18].

Adolescence represents a critical life phase for mental health [19,20], but it also can be considered a developmental transition phase [21] with great opportunities for improving health [18] in which adolescents are particularly receptive to educational interventions to promote MHL. Cairns and Rossetto (2019) reinforce that MHL supports children, adolescents, and young people to attain positive mental health and wellbeing, as well as facilitating timely access to appropriate help when mental health problems occur [22]. 

In Portugal, the Health Literacy Action Plan 2019–2021 [23], referring to HL as an opportunity to promote health throughout the life cycle taking into account specificities of each stage of development, reinforces the need for interventions that promote adolescents’ HL, namely, in the school context, where adolescents spend most of their time. The Portugal School Health Program 2015 [24] presents the promotion of HL as a general objective and anxiety as an area of intervention in adolescence.

Psychoeducation emerges as a useful educational strategy often associated with prevention and intervention in mental illness, but it is also emerging in the promotion of mental health and MHL, particularly in the school context [25,26,27,28,29,30,31]. However, some studies still reported limitations and suggestions for future research [26,28,30,31,32,33].

In Portugal, the Manual of Good Practices in Health Literacy: Training of Health Professionals [34] reinforces the importance of psychoeducation as a tool in health literacy promotion. According to Portuguese regulations, mental health and psychiatric nurses assist the person throughout the life cycle, family, groups, and community in optimizing mental health and develop psychoeducational interventions. They also mobilize the context and individual, family, or group dynamics of the community [35] and promote MHL as one of the quality standards of mental health and psychiatric nursing care [36].

The ProLiSMental psychoeducational intervention to promote adolescents’ MHL on anxiety at a school context was developed [37,38,39,40,41,42]. The purpose of this article is to present the results of a quasi-cluster randomized controlled trial used to pilot the ProLiSMental psychoeducational intervention, focused on knowledge, skills, and behavioral intentions of the following MHL components: (a) anxiety recognition; (b) anxiety prevention strategies; (c) anxiety self-help strategies.

## 2. Materials and Methods

Medical Research Council Framework [43,44] was used for the development of the ProLiSMental psychoeducational intervention as a complex intervention, throughout development and feasibility stages. Several previous studies were performed in this area with different aims: to identify the evidence, with a systematic review [37]; to identify the theory, with a narrative review [38]; to model processes and results, with adolescents’ and education/health professionals’ focus groups [39] and experts’ e-Delphi [40]; to test the design and the methodology and assess the acceptability and feasibility, with an exploratory study [41,42]. A pilot study was followed, a small-scale study to assess the primary outcome of a future full-scale study [45,46]. Some authors have referred to the pertinence of using cluster randomized trials to pilot and evaluate complex interventions [47,48]. In this study, a pilot quasi-cluster randomized controlled trial, with pretest, posttest, and one-month follow-up design was used. This study is registered in www.clinicaltrials.org (accessed on 28 July 2021) with ID number NCT03872817.

### 2.1. Participants

Adolescents in the 9th grade from a school in the Central Region of Portugal were eligible to participate in the pilot study of the ProLiSMental psychoeducational intervention. According to exception criteria, they were excluded from the study if being students with special educational needs (*N* = 1); students who participated in the feasibility study of this intervention (*N* = 11), and students and legal guardians who did not accept to participate and did not sign the informed consent (*N* = 1). The class directors recruited eligible participants.

**Figure 1:**
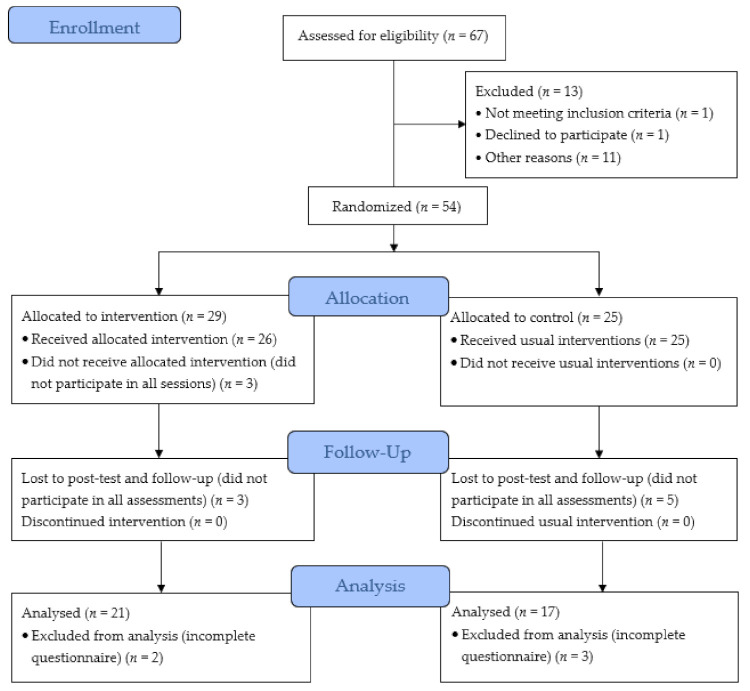
Participants’ flow diagram through the phases of the quasi-cluster randomized controlled trial.

We have followed the authors’ suggestion [45] of using a 95% confidence interval approach to estimate sample size. From the total number of 67 adolescents attending the 9th grade at that school in the central region of Portugal, using G*Power 3.1 [49], with a 95% confidence level and a sampling error of 5%, the sample size recommended for this study was 58 adolescents. Only 54 adolescents and their legal guardians from two classes in this school met the inclusion criteria, corresponding to a confidence level of 90% and a sampling error of 5%. After class randomization, 29 adolescents were included in the intervention group (IG), which received the ProLiSMental psychoeducational intervention first, and 25 adolescents in the waiting list control group (WLCG), which received the same intervention after it was completed in the IG. However, of these only 38 adolescents participated in all sessions of the ProLiSMental psychoeducational intervention and all phases of data collection, with 21 (55.3%) adolescents in the IG and 17 (44.7%) adolescents in the WLCG, which represents a 70.4% retention rate (Figure 1).

### 2.2. Intervention

Based on the theoretical framework of several authors [13,14,16,21,50,51,52,53,54], the ProLiSMental psychoeducational intervention to promote adolescents’ MHL on anxiety was developed. Its main objective was to enable adolescents in the school context to access, understand, evaluate, and apply information about mental health, to promote and maintain good mental health, and to facilitate anxiety recognition, prevention, and management.

The ProLiSMental psychoeducational intervention is structured in phases [42], whose total number of sessions can vary between 4 and 13 sessions and the total duration between 6 h and 13 h and 30 min. The psychoeducational phase with adolescents consists of four weekly sessions of 90 min or eight weekly sessions of 45 min. In each session, the following topics are approached: (1) “Recognise”: mental health, emotions, and anxiety in adolescents”; (2) “Take care”: mental health promotion and anxiety prevention, management, and self-help strategies; (3) “Seek help”: mental health first aid actions, and seeking informal and formal help, including specialized health professionals; (4) “Action!”: from knowledge on mental health and anxiety prevention, and management to action in everyday life.

In this pilot study, the ProLiSMental psychoeducational intervention was delivered in the school context by the principal researcher and three specialists nurses from primary health care (specializing in mental health and psychiatric nursing; child health and pediatric nursing or community health nursing) with experience in school health nursing and with previous 10-h training on the ProLiSMental psychoeducational intervention. In all sessions, three trainers, the researcher, and two of three primary health care nurses were always present, one of whom assumed the role of a non-participating observer.

### 2.3. Outcomes Measures 

The QuALiSMental [55], the European Portuguese version of the “Survey of Mental Health Literacy in Young People—Interview Version” [20], was used in this quasi-cluster randomized controlled trial to measure adolescents’ MHL. The first part of the questionnaire includes instructions for filling out and questions of sociodemographic characterization of adolescents (gender, age, with whom they live, educational background, and employment situation of legal guardians). The second part reports a situation of an adolescent’s social anxiety [41], followed by questions regarding the different components of the MHL concept [14] from the perspective of knowledge, skills, and behavioral intentions [51]: (a) anxiety recognition; (b) anxiety prevention strategies; (c) anxiety self-help strategies; (d) help-seeking options; (e) anxiety mental health first aid. In this article, we will focus on the results for these first three components.

### 2.4. Data Collection and Analysis

Each class was allocated to the IG or the WLCG with single-blinded randomization by tossing a coin before the first assessment has been taken. The IG received the ProLiSMental psychoeducational intervention in April 2016, and three assessments were carried out with the collaboration of the teachers with the role of class directors, differentiating from the facilitators of the ProLiSMental psychoeducational intervention: (1) one day before (pretest); (2) one day after (posttest); (3) one month after (follow-up). After the ProLiSMental psychoeducational intervention was completed in the IG, the WLCG also received the same intervention in May 2016. In the WLCG, the three assessments took place: (1) one month before; (2) one day before; (3) one day after the intervention.

The statistical analysis was performed using the IBM-SPSS 27.0 software. After the inversion of three dimensions (“avoid stressful situations”; “drink alcohol to relax”; and “smoke to relax”), appropriate descriptive statistics (e.g., mean, standard deviation), and absolute and percentage frequencies were calculated, where appropriate. The Cochran Q test with Dunn’s post-hoc procedures, adjusted with Bonferroni’s significance correction with a *p*-value of 0.05 was used. For the Cochran Q test, the chance-corrected measure of effect size (R) was used, where R is zero under chance conditions, unity when agreement among the *n* subjects is perfect, and negative under conditions of disagreement [56]. In this study, we considered the effect size intervals: up to 0.2—small; 0.2 to 0.5—moderate; 0.6 to 0.8—relatively large; 0.8 to 1—large. To assess the association between the IG and the WLCG at each moment, the chi-square test (χ^2^) was used.

### 2.5. Ethical Procedures

This study was approved by several organizations: (a) the Ethics Committee of the Regional Health Administration of Central Portugal (Administração Regional de Saúde do Centro), number 71/2015; (b) the National Data Protection Commission of Portugal (Comissão Nacional de Proteção de Dados), number 10880/2015; (c) General Direction of Education (Direção Geral da Educação), number 0506400001/2015; (d) Executive Board of the basic and secondary school where the study was carried out. Ethical principles of the revised Helsinki Declaration [57] (Association, 2013) were adopted and both adolescents and their legal guardians signed and delivered an informed consent form, which highlighted the voluntary participation and confidentiality ethical considerations.

## 3. Results

### 3.1. Sociodemographic Characterization 

Of the 38 participants in this study, 24 (63.2%) were female and 14 (36.8%) were male, with an average age of 14.50 years (SD = 0.89). In the baseline, the results regarding the sociodemographic characteristics of the participants in the IG (*n* = 21) and the WLCG (*n* = 17) (Table 1) show the groups were homogeneous (*p* > 0.05).

### 3.2. Anxiety Recognition 

Regarding the “anxiety recognition” component, Table 2 shows the findings in the three assessments.

In the IG after the intervention, “anxiety” was significantly recognized, with moderate effect size, by the participants (Q = 26.143; *p* < 0.001; R = 0.396), with a significant association between the moments a-b and a-c (*p* < 0.001). There were also statistically significant differences in the dimensions: “social anxiety” (Q = 10.429; *p* < 0.01; R = 0.132, with a significant association between the moment a-b and a-c, *p* < 0.01) and “shyness” (Q = 19.625; *p* < 0.001; R = 0.318, with a significant association between the moment a-b and a-c, *p* < 0.001).

In the WLCG, the third assessment also shows significant differences, with moderate effect size, in the dimensions: “anxiety” (Q = 22.154; *p* < 0.001; R = 0.434, with a significant association between the moment a-c and b-c, *p* < 0.001) and “social anxiety” (Q = 12.000; *p* < 0.01; R = 0.233, with a significant association between the moment a-c and b-c, *p* < 0.01).

In the analysis of independence between the groups at each moment, in this component there was a significant association between the IG and WLCG and the dimensions: “anxiety” (χ^2^ = 20.442; *p* < 0.001) and “social anxiety” (χ^2^ = 10.986; *p* < 0.001) in the second assessment. 

Although about 47.6% and 70.6% of participants in the IG and WLCG groups respectively mentioned the “fear of exposure” in the baseline, in the second and the third assessment this percentage decreased statistically significant for the WLCG (Q = 12.200; *p* < 0.01; R = 0.163, with a significant association between the moments a-c, *p* < 0.001).

### 3.3. Anxiety Prevention Strategies 

In the “anxiety prevention strategies” component (Table 3), there were higher percentages of correct answers of the participants in the baseline for both groups, except for the dimensions “do not avoid stressful situations” and “have a religious or spiritual belief”. In the first, there were no changes after the intervention for both groups. In the second, there was an improvement in the IG in the posttest and follow-up.

In the baseline, the highest percentages of correct answers were found in dimensions “regular contact with friends”, “regular contact with family”, and “improve self-esteem” for IG, which increased in the posttest. Particularly, to the dimension “regular contact with family”, in the second assessment, there was a significant association to the WLCG (χ^2^ = 5.828; *p* < 0.05).

In the IG, after the intervention, there were statistically significant differences in the dimensions: “exercise regularly” (Q = 8.000; *p* < 0.01; R = 0.062, with a significant association between the moment a-b and a-c, *p* < 0.05). There were also significant differences in “regular sleep habits” (Q = 6.500; *p* < 0.05; R = 0.042, with a significant association between the moments a-c, *p* < 0.05); “regular relaxation training” (Q = 8.000; *p* < 0.05; R = 0.063, with a significant association between the moments a-b and a-c, *p* < 0.05). The dimension “have a religious or spiritual belief” (Q = 12.250; *p* < 0.01; R = 0.093, had a significant association between the moments a-b, *p* < 0.01), reinforcing improvements after the intervention. 

In the WLCG, after the intervention, differently from the IG, the dimensions “do not drink alcohol” (Q = 6.000; *p* < 0.05; R = 0.027) and “healthy eating” (Q = 7.000; *p* < 0.05; R = 0.082) evidence a significant association between the moments a-c (*p* < 0.05).

### 3.4. Anxiety Self-Help Strategies 

Regarding the “anxiety self-help strategies” component (Table 4), in the baseline, the highest percentages of correct answers of the participants were found in the dimensions “exercise regularly”, “do not drink alcohol to relax”, and “do not smoke to relax”. These dimensions remained or increased in the posttest and follow-up for the IG and remained after the intervention for the WLCG, not showing statistically significant differences in the three moments of assessment.

In the IG, this component was the one that showed statistically significant differences after the intervention in more dimensions. We found a significant association between the moment a-b and a-c. Found however small association in relatively large effect sizes, such as “regular relaxation training” (Q = 10.000; *p* < 0.01; R = 0.093); “meditation regularly” (Q = 16.000; *p* < 0.001; R = 0.201); “acupuncture” (Q = 34.000; *p* < 0.001; R = 0.614). Also in the dimensions “get up early and sunbathe” (Q = 14.000; *p* < 0.01; R = 0.222); “website with credible information” (Q = 11.400; *p* < 0.01; R = 0.099); “self-help book” (Q = 8.769; *p* < 0.05; R = 0.093); “join a support group” (Q = 18.000; *p* < 0.001; R = 0.157), and “seek specialized mental health help” (Q = 13.273; *p* < 0.01; R = 0.152).

In the analysis of independence between the groups at each moment, there was a significant association between the IG and WLCG in the second assessment. These differences were identified in the dimensions “meditation regularly” (χ^2^ = 9.299; *p* < 0.01); “acupuncture” (χ^2^ = 23.543; *p* < 0.001); “get up early and sunbathe” (χ^2^ = 10.600; *p* < 0.01); “therapy with a specialized professional” (χ^2^ = 4.795; *p* < 0.05); “website with credible information” (χ^2^ = 7.012; *p* < 0.01); “self-help book” (χ^2^ = 4.962; *p* < 0.05) and “seek specialized mental health help” (χ^2^ = 10.264; *p* < 0.001). These data showed improvements after the intervention. The significant association between the IG and WLCG continues in the third assessment in the dimensions: “acupuncture” (χ^2^ = 5.208; *p* < 0.05) and “get up early and sunbathe” (χ^2^ = 5.522; *p* < 0.05). This means that despite the increase of the correct answers in these dimensions in the WLCG, they were not enough to approach the values obtained in the IG after the intervention.

Additionally, in the WLCG after the intervention, differently from the IG, there were also significant improvements in the “therapy with a specialized professional” dimension (Q = 9.556; *p* < 0.01; R = 0.110), with a significant association between the moments a-c and b-c (*p* < 0.05).

## 4. Discussion

This pilot study of the ProLiSMental psychoeducational intervention assessed the primary outcome of the future full-scale trial: improvement of adolescents’ knowledge, skills, and behavioral intentions in the following components of anxiety mental health literacy at a school context: (a) anxiety recognition; (b) anxiety prevention strategies; (c) anxiety self-help strategies. 

Regarding the first component, there was a significant improvement with a moderate effect size in the recognition of anxiety as a normative emotion and the recognition of pathological anxiety, namely the signs and symptoms of social anxiety. These results are in line with other studies on the development of mental health literacy interventions [58,59,60,61,62,63]. There was also a reduction in the percentage of responses after intervention in the dimensions “shyness” and “fear of exposure”, that despite its frequent association with social anxiety, recent evidence clarifies its similarities and differences [64,65,66]. 

It is positive that the adolescents present good results in the anxiety prevention strategies component in the baseline. However, after the intervention, there were significant improvements with small effect size, in the dimensions: “exercise regularly”; “regular sleep habits”; “regular relaxation training”; “have a religious or spiritual belief” in the IG, and “do not drink alcohol” and “healthy eating” in the WLCG. These results corroborate the importance of healthy lifestyles and coping strategies for promoting mental health and well-being and preventing pathological anxiety from the earliest age groups such as children and adolescents [67,68]. These results reinforce the need for interventions to promote mental health and MHL on these topics [18,27,29,69]. However, the results of the dimension “do not avoid stressful situations” as an emotion regulation strategy [70,71] decreased instead of increasing, throughout the three assessments, which suggest attention in the future full-scale trial. We wonder if this is due to any lack of clarification by facilitators or difficulty understanding by adolescents during the ProLiSMental psychoeducational intervention or when filling out the QuALiSMental questionnaire since this dimension appeared inverted.

In the “anxiety self-help strategies” component, some dimensions related to healthy lifestyles also showed high percentages in the baseline: “exercise regularly”; “do not drink alcohol to relax”; and “do not smoke to relax”. Another dimension related to healthy lifestyles is “get up early and sunbathe”, which showed statistically significant differences with moderate effect size in the IG after the intervention. There were other statistically significant improvements, with small to relatively large effect sizes, in many dimensions: “regular relaxation training”; “meditation regularly”; “acupuncture”; “website with credible information”; “self-help book”; “join a support group”; and “seek specialized mental health help” in the IG and “therapy with a specialized professional” in the WLCG. These results reinforce the importance of the positive view of the use of self-help strategies by young people, showing interest in learning new forms of self-help as a way of coping with anxiety [14,58]. Similar results regarding the “self-help strategies” component are reported in other studies with interventions to promote MHL in adolescents [58,59,60,61,62,63], despite the differences between them in terms of the content and methodology of the interventions, for example, the components of the MHL under study, the number of sessions, the facilitators and the recipients. 

Given the current global context of the Covid 19 pandemic, an emerging suggestion of this study is the possibility of developing ProLiSMental psychoeducational intervention in a digital and online way as noted by a recent systematic review [72]. 

This intervention promotes adolescents’ MHL as a protective factor of mental health in a universal way [50] and because we believe that simultaneously it contributes to the prevention of pathological anxiety, we suggest in future studies, in addition to MHL assessment, the possibility of assessing adolescents’ anxiety levels before, after the intervention and over the time. We also suggest assessing satisfaction with the ProLiSMental psychoeducational intervention implementation, with validated instruments and even including legal guardians and teachers, since there are some sessions aimed at them. Reinforcing this idea, we also suggest future studies for the development of psychoeducational interventions to promote anxiety MHL aimed at these recipients.

Another interesting suggestion is the possibility of extending the ProLiSMental psychoeducational intervention to other contexts, from universal to a selective and indicated prevention perspective, reinforcing its potential to be developed throughout the mental health continuum [50].

Considering the advantages of cluster randomized trials [47,48], we recognize the limitations related to the small sample size in this study, conditioning the retention and the randomization process, which increased the risk of bias. 

For this reason, we suggest increasing the sample size in the full-scale trial, recruiting more participants, on the one hand, and increasing the number of facilitators, on the other hand, providing training on the ProLiSMental psychoeducational intervention, that will allow replication of the intervention on a larger scale.

The common areas of methodological uncertainty like recruitment, randomization, retention, blinding and data collection/outcome assessment methods [45,46] were tested by this pilot study. Considering its limitations and suggestions, the results of this pilot study allow us to proceed to a full-scale trial, without changes in the components of the complex ProLiSMental psychoeducational intervention.

## 5. Conclusions

This pilot quasi-cluster randomized controlled trial shows the improvement of adolescents’ knowledge, skills, and behavioral intentions after the ProLiSMental psychoeducational intervention, in the components of anxiety MHL: (a) anxiety recognition; (b) anxiety prevention strategies; (c) anxiety self-help strategies. Overall, there was a significant improvement and small to relatively strong effect size in many dimensions of anxiety MHL components in the IG compared to the WLCG, considering the evolution throughout the three assessments. Was similar in the WLCG at the third assessment, after the ProLiSMental psychoeducational intervention.

We highlight the significant improvements after the intervention and follow-up. These improvements include the recognition of normal and pathological anxiety; the anxiety prevention strategies: “exercise regularly”; “regular sleep habits”; “regular relaxation training”; “have a religious or spiritual belief” in the IG. Also the self-help strategies as a way of coping with anxiety: “get up early and sunbathe”; “regular relaxation training”; “meditation regularly”; “acupuncture”; “website with credible information”; “self-help book”; “join a support group”; and “seek specialized mental health help” in the IG.

The ProLiSMental psychoeducational intervention showed improvements of knowledge, skills, and behavioral intentions in various components of anxiety MHL as predictors of behaviors that promote mental health and prevent pathological anxiety, reinforcing the progression to the full-scale trial.

Despite the development, the feasibility evaluation, and the pilot of ProLiSMental psychoeducational intervention began before the Covid 19 pandemic context, due to the increased levels of anxiety in the general population and adolescents, in particular, this intervention seems to be extremely useful.

We believe that mental health and psychiatric nurses according to their professional and legal framework, as members of transdisciplinary teams, can contribute in a solid and scientifically sustained way to the development and implementation of psychoeducational interventions to promote MHL, mental health, and anxiety prevention, and simultaneously, to empower adolescents in schools.

Mental health and psychiatric nurses have an important role in facilitating networking between different health care professionals and contexts, with important contributions in the identification and early intervention in mental health problems in children and adolescents. 

## Figures and Tables

**Table 1:** Participants’ sociodemographic characteristics in the baseline (*N* = 38).

Variable		IG (*n* = 21)	WLCG (*n* = 17)	*p* (Effect Size)
Age in years, M (SD) *		14.76 (1.9)	14.18 (0.39)	0.089 (0.076) ^a^
Female, *n* (%)		13 (61.9)	11 (64.7)	0.859 (0.029) ^b^
Adolescents live with, *n* (%)	Parents	21 (100)	17 (100)	†
Father’s education, *n* (%)	1st cycle of schooling	1 (4.8)	1 (5.9)	0.755 (0.219) ^c^
	3rd cycle of schooling	5 (23.8)	5 (29.4)	
	High school	6 (28.6)	2 (11.8)	
	Bachelor’s degree	4 (28.6)	5 (29.4)	
	Master’s/doctorate	5 (23.8)	4 (23.5)	
Mother’s education, *n* (%)	1st cycle of schooling	1 (4.8)	0 (0)	0.108 (0.447) ^c^
	2nd cycle of schooling	0 (0)	1 (5.9)	
	3rd cycle of schooling	2 (9.5)	2 (11.8)	
	High school	8 (38.1)	1 (5.9)	
	Bachelor’s degree	6 (28.6)	7 (41.2)	
	Master’s/doctorate	4 (19.0)	6 (35.3)	
Father’s professional situation, *n* (%)	Employeed	20 (95.2)	16 (94.1)	0.543 (0.149) ^c^
Mother’s professional situation, *n* (%)	Employeed	21 (100)	17 (100)	†

* Mean (M), Standard Deviation (SD); ^a^ Mann Whitney *p*-value (η^2^); ^b^ Chi-square *p*-value (φ); ^c^ L-R *p*-value (V); † Test not performed or measure not calculated.

**Table 2:** Findings for “anxiety recognition” in the three assessments by group.

Dimension		a	b	c	Q ^e^	R ^f^	Post-Hoc ^g^		
		*n* (%)	*n* (%)	*n* (%)			a-b	a-c	b-c
Anxiety	IG	5 (23.8)	19 (90.5) ^d^	18 (85.7)	26.143 ***	0.396	***	***	ns
	WLCG	3 (17.6)	3 (17.6) ^d^	15 (88.2)	22.154 ***	0.434	ns	***	***
Social anxiety	IG	2 (9.5)	9 (42.9) ^d^	11 (52.4)	10.429 **	0.132	**	**	ns
	WLCG	0 (0.0)	0 (0.0) ^d^	6 (35.3)	12.000 **	0.233	ns	**	**
Fear of exposure	IG	10 (47.6)	6 (28.6)	5 (23.8)	3.500 ^ns^	0.021	‡	‡	‡
	WLCG	12 (70.6)	8 (47.1)	3 (17.6)	12.200 **	0.163	ns	***	ns
Shyness	IG	16 (76.2) ^d^	3 (14.3)	4 (19.0)	19.625 ***	0.318	***	***	ns
	WLCG	5 (29.4) ^d^	5 (29.4)	5 (29.4)	<0.001 ^ns^	−0.027	‡	‡	‡
Low self-esteem	IG	3 (14.3)	0 (0.0)	2 (9.5)	3.500 ^ns^	0.021	‡	‡	‡
	WLCG	4 (23.5)	2 (11.8)	0 (0.0)	4.800 ^ns^	0.054	‡	‡	‡
Developmental problem	IG	3 (14.3)	0 (0.0)	1 (4.8)	3.500 ^ns^	0.026	‡	‡	‡
	WLCG	0 (0.0)	2 (11.8)	1 (5.9)	3.000 ^ns^	0.014	‡	‡	‡
Depression	IG	0 (0.0)	0 (0.0)	0 (0.0)	†	†	‡	‡	‡
	WLCG	0 (0.0)	2 (11.8)	1 (5.9)	2.000 ^ns^	<0.001	‡	‡	‡

(a) First assessment; (b) second assessment; (c) third assessment; ^d^ significance associated with the Chi-Square test (χ^2^) between group and dimension; ^e^ comparison with Cochran’s Q test (Q); ^f^ chance-corrected measures of effect size (R); ^g^ post-hoc procedures with Dunn’s test, adjusted with Bonferroni’s significance correction; ** *p* < 0.01; *** *p* < 0.001; ns = not significant; † test not performed or measure not calculated; ‡ post-hoc procedure not calculated.

**Table 3:** Findings for “anxiety prevention strategies” in the three assessments by group.

Dimension		a	b	c	Q ^e^	R ^f^	Post-Hoc ^g^		
		*n* (%)	*n* (%)	*n* (%)			a-b	a-c	b-c
Exercise regularly	IG	16 (76.2)	20 (95.2)	20 (95.2)	8.000 **	0.062	*	*	ns
	WLCG	11 (64.7)	15 (88.2)	16 (94.1)	7.000 *	0.082	ns	*	ns
Do not avoid stressful situations	IG	3 (14.3)	2 (9.5)	1 (4.8)	2.000 ^ns^	<0.001	‡	‡	‡
	WLCG	2 (11.8)	2 (11.8)	1 (5.9)	1.000 ^ns^	−0.009	‡	‡	‡
Regular contact with friends	IG	19 (90.5)	20 (95.2)	19 (90.5)	1.000 ^ns^	−0.007	‡	‡	‡
	WLCG	15 (88.2)	13 (76.5)	15 (88.2)	4.000 ^ns^	0.012	‡	‡	‡
Regular contact with family	IG	19 (90.5)	20 (95.2) ^d^	19 (90.5)	1.000 ^ns^	−0.007	‡	‡	‡
	WLCG	12 (70.6)	11 (64.7) ^d^	13 (76.5)	1.500 ^ns^	−0.004	‡	‡	‡
Do not use drugs	IG	18 (85.7)	18 (85.7)	18 (85.7)	<0.001 ^ns^	−0.017	‡	‡	‡
	WLCG	14 (82.4)	15 (88.2)	16 (94.1)	1.500 ^ns^	−0.008	‡	‡	‡
Regular sleep habits	IG	16 (76.2)	19 (90.5)	20 (95.2)	6.500 *	0.042	ns	*	ns
	WLCG	12 (70.6)	12 (70.6)	16 (94.1)	6.400 *	0.051	ns	ns	ns
Do not drink alcohol	IG	16 (76.2)	18 (85.7)	17 (81)	0.857 ^ns^	−0.013	‡	‡	‡
	WLCG	9 (52.9)	11 (64.7)	13 (76.5)	6.000 *	0.027	ns	*	ns
Healthy eating	IG	17 (81.0)	19 (90.5)	20 (95.2)	4,667 ^ns^	0.021	‡	‡	‡
	WLCG	12 (70.6)	13 (76.5)	17 (100)	7.000 *	0.082	ns	*	ns
Regular relaxation training	IG	16 (76.2)	20 (95.2)	20 (95.2)	8.000 *	0.063	*	*	ns
	WLCG	13 (76.5)	13 (76.5)	14 (82.4)	0.286 ^ns^	−0.028	‡	‡	‡
Improve self-esteem	IG	19 (90.5)	20 (95.2)	20 (95.2)	2.000 ^ns^	<0.001	‡	‡	‡
	WLCG	17 (100)	17 (100)	16 (94.1)	2.000 ^ns^	<0.001	‡	‡	‡
Have a religious or spiritual belief	IG	3 (14.3)	11 (52.4) ^d^	8 (38.1)	12.250 **	0.093	**	ns	ns
	WLCG	2 (11.8)	3 (17.6) ^d^	4 (23.5)	1.500 ^ns^	−0.005	‡	‡	‡

(a) First assessment; (b) second assessment; (c) third assessment; ^d^ significance associated with the Chi-Square test (χ^2^) between group and dimension; ^e^ comparison with Cochran’s Q test (Q); ^f^ chance-corrected measures of effect size (R); ^g^ post-hoc procedures with Dunn’s test, adjusted with Bonferroni’s significance correction; * *p* < 0.05; ** *p* < 0.01; ns = not significant; ‡ post-hoc procedure not calculated.

**Table 4:** Findings for “anxiety self-help strategies” in the three assessments by group.

Dimension		a	b	c	Q ^e^	R ^f^	Post-Hoc ^g^		
		*n* (%)	*n* (%)	*n* (%)			a-b	a-c	b-c
Exercise regularly	IG	19 (90.5)	20 (95.2)	19 (90.5)	1.000 ^ns^	−0.007	‡	‡	‡
	WLCG	14 (82.4)	14 (82.4)	16 (94.1)	2.000 ^ns^	<0.001	‡	‡	‡
Regular relaxation training	IG	15 (71.4)	20 (95.2)	20 (95.2)	10.000 **	0.093	*	*	ns
	WLCG	14 (82.4)	15 (88.2)	17 (100.0)	3.500 ^ns^	0.027	‡	‡	‡
Meditation regularly	IG	12 (57.1)	20 (95.2) ^d^	20 (95.2)	16.000 ***	0.201	**	**	ns
	WLCG	11 (64.7)	9 (52.9) ^d^	14 (82.4)	3.800 ^ns^	0.032	‡	‡	‡
Acupuncture	IG	2 (9.5)	19 (90.5) ^d^	19 (90.5) ^d^	34.000 ***	0.614	***	***	ns
	WLCG	3 (17.6)	2 (11.8) ^d^	10 (58.8) ^d^	14.250 **	0.187	ns	**	**
Get up early and sunbathe	IG	14 (66.7)	21 (100.0) ^d^	21 (100.0) ^d^	14.000 **	0.222	**	**	ns
	WLCG	6 (35.3)	10 (58.8) ^d^	13 (76.5) ^d^	6.727 *	0.084	ns	*	ns
Therapy with a specialized professional	IG	14 (66.7)	17 (81.0) ^d^	19 (90.5)	4.222 ^ns^	0.032	‡	‡	‡
	WLCG	7 (41.2)	8 (47.1) ^d^	14 (82.4)	9.556 **	0.110	ns	*	*
Website with credible information	IG	7 (33.3)	14 (66.7) ^d^	15 (71.4)	11.400 **	0.099	*	**	ns
	WLCG	4 (23.5)	4 (23.5) ^d^	10 (58.8)	8.000 *	0.094	ns	*	*
Self-help book	IG	7 (33.3)	15 (71.4) ^d^	14 (66.7)	8.769 *	0.093	*	ns	ns
	WLCG	8 (47.1)	6 (35.3) ^d^	9 (52.9)	1.167 ^ns^	−0.016	‡	‡	‡
Join a support group	IG	7 (33.3)	16 (76.2)	16 (76.2)	18.000 ***	0.157	***	***	ns
	CG	8 (47.1)	8 (47.1)	12 (70.6)	6.400 *	0.035	ns	ns	ns
Seek specialized mental health help	IG	9 (42.9)	18 (85.7) ^d^	17 (81.0)	13.273 **	0.152	**	**	ns
	CG	11 (64.7)	6 (35.3) ^d^	13 (76.5)	11.143 **	0.104	ns	ns	**
Do not drink alcohol to relax	IG	21 (100.0)	21 (100.0)	21 (100.0)	†	†	‡	‡	‡
	CG	16 (94.1)	15 (88.2)	15 (88.2)	2.000 ^ns^	<0.001	ns	ns	ns
Do not smoke to relax	IG	19 (90.5)	21 (100.0)	21 (100.0)	4.000 ^ns^	0.034	ns	ns	ns
	CG	16 (94.1)	16 (94.1)	17 (100.0)	1.000 ^ns^	−0.021	ns	ns	ns

(a) First assessment; (b) second assessment; (c) third assessment; ^d^ significance associated with the Chi-Square test (χ^2^) between group and dimension; ^e^ comparison with Cochran’s Q test (Q); ^f^ chance-corrected measures of effect size (R); ^g^ post-hoc procedures with Dunn’s test, adjusted with Bonferroni’s significance correction; * *p* < 0.05; ** *p* < 0.01; *** *p* < 0.001; ns = not significant; † test not performed or measure not calculated; ‡ post-hoc procedure not calculated.

## Clinical Trial Registration Information

ClinicalTrials.gov ID: NCT03872817.
